# Active microorganisms thrive among extremely diverse communities in cloud water

**DOI:** 10.1371/journal.pone.0182869

**Published:** 2017-08-08

**Authors:** Pierre Amato, Muriel Joly, Ludovic Besaury, Anne Oudart, Najwa Taib, Anne I. Moné, Laurent Deguillaume, Anne-Marie Delort, Didier Debroas

**Affiliations:** 1 Université Clermont Auvergne, CNRS, Institut de Chimie de Clermont-Ferrand, Clermont-Ferrand, France; 2 Université Clermont Auvergne, CNRS, Laboratoire Microorganismes: Génome et Environnement, Clermont-Ferrand, France; 3 Université Clermont Auvergne, CNRS, Observatoire de Physique du Globe, Clermont-Ferrand, France; Wilfrid Laurier University, CANADA

## Abstract

Clouds are key components in Earth’s functioning. In addition of acting as obstacles to light radiations and chemical reactors, they are possible atmospheric oases for airborne microorganisms, providing water, nutrients and paths to the ground. Microbial activity was previously detected in clouds, but the microbial community that is active *in situ* remains unknown. Here, microbial communities in cloud water collected at puy de Dôme Mountain’s meteorological station (1465 m altitude, France) were fixed upon sampling and examined by high-throughput sequencing from DNA and RNA extracts, so as to identify active species among community members. Communities consisted of ~10^3^−10^4^ bacteria and archaea mL^-1^ and ~10^2^−10^3^ eukaryote cells mL^-1^. They appeared extremely rich, with more than 28 000 distinct species detected in bacteria and 2 600 in eukaryotes. Proteobacteria and Bacteroidetes largely dominated in bacteria, while eukaryotes were essentially distributed among Fungi, Stramenopiles and Alveolata. Within these complex communities, the active members of cloud microbiota were identified as Alpha- (Sphingomonadales, Rhodospirillales and Rhizobiales), Beta- (Burkholderiales) and Gamma-Proteobacteria (Pseudomonadales). These groups of bacteria usually classified as epiphytic are probably the best candidates for interfering with abiotic chemical processes in clouds, and the most prone to successful aerial dispersion.

## 1. Introduction

The atmospheric envelope is a fundamental component of Earth’s functioning. Apart from holding huge energy exchanges, it transports, transforms and redistributes material at a large scale; it also participates to the spreading of microorganisms over the globe (e.g., [1–5]). Outdoor, the air is dotted with microorganisms (virus, bacteria, archaea, and eukaryotes) originating from surface habitats like vegetation, soil, water, or Humans/animals among natural sources [6–8], at concentrations varying from ~10^2^ to ~10^6^ cells m^-3^ (e.g., [9,10]. Some of them are regarded with attention for potential health hazards to Humans, animals and plants [11]. Surface ecosystems, also, are exposed to the continuous flow of diverse microbial incomers deposited from the atmosphere, bringing competitors, genetic material, and early colonizers in emerging habitats (e.g., [3,12]. Yet, environmental fitness tends to decrease with increasing distance from the source as habitats diverge [13], while, in addition, atmospheric transport exposes cells to harsh environmental conditions [14,15]. Hence, for microorganisms unable to produce resistance forms (spores), maintaining metabolic activity appears decisive for survival and possibility of successful establishment in the receptacle environment (e.g. [16].

Within the atmospheric system, clouds are genuine atmospheric interfaces with the ground: they physically connect high altitudes with the surface by being to a large extent at the origin of wet deposition of aerosols, including microorganisms [1,17,18]. Cloud water is a complex mixture of soluble gas and particles dissolved into millions of micron-sized water droplets, and forming very reactive and dynamic systems (e.g., [19]. As non-soluble biological particles, some microorganisms can physically impact clouds by acting as embryos for the formation of water droplets and ice crystals [20,21], with subsequent impacts on hydrological cycles [22–26]. Observations of microbiological features in fog and clouds raised the possibility that these also represent habitats for microorganisms [27–29], where they would actively take part in the chemical reactivity through metabolic activity and nutrient utilization [30–34]. So far these active « inhabitants » of clouds remain largely unknown. Microbiological studies in the atmosphere, including precipitation and deposition dust, essentially focused on the biodiversity, pathogens, emission sources and environmental drivers [3,6,7,10,35]. A predominance of Gram-negative bacteria (Alpha-, Beta- and Gamma-Proteobacteria, Bacteroidetes) is often observed, and attributed to inputs from soil and plants, with high temporal and spatial variability [3,6]. Current knowledge about the microorganisms living in clouds is essentially based on cultures approaches, so limited to a small fraction (< 1%) of the whole community. These indicated the presence bacteria and fungi, with prevailing groups, in Proteobacteria notably, and others appearing only once in a while [8,36–38]. Interrogations concerning the actual *in situ* functioning of microbial communities in clouds remain, starting with the identification of active members. Yet, these are probably better equipped than others (or better fitted) for surviving in the atmosphere and clouds [14,15,39], interfering with abiotic atmospheric processes, and they likely represent the potential successful colonizers of distant habitats. Here, using molecular methods, we investigate the structure of cloud water microbial communities and clarify our current vision of clouds as habitats by identifying active members. This consortium of active microorganisms finally revealed provides crucial information for further research on the interactions existing between microbial communities and abiotic processes in clouds, as well as important insights into the aerial dispersion of microorganisms.

## 2. Material and methods

### 2.1. Sample collection

Three cloud water samples were collected during the fall 2013 from the atmospheric station at the summit of puy de Dôme Mountain (1465 m a.s.l., 45.772° N, 2.9655° E, France). Specific permission was not required since the station is operated by OPGC (Observatory of the Globe of Clermont-ferrand), who collaborated this study. The field study carried out did not involve any endangered of protected species. Samples were collected at an air flow rate of 108 m^3^ h^-1^ using a cloud droplet impactor similar as in [24,30,40]. It has been slightly modified for allowing immediate fixation of the biological content (DNA and RNA) upon collection using a fixative agent: the water collected was transferred continuously, by gravity through autoclaved silicone tubing, to a sterile glass bottle containing 200 mL of a saturated ammonium sulphate solution used as surrogate for commercial fixative agent (i.e. RNA *Later*). This later was prepared under sterile conditions from fresh powders dissolved into sterile deionized water, then stored in sterile bottles. When samples froze upon impaction in the sampler, the ice collected was immediately melted into 200 mL of cold fixative solution. Before each sampling occasion, the presence of contaminants along the sampling apparatus and in the fixative solution was controlled by pooring 200 mL of sterile water into the sampler. The resulting 400 mL control sample was then processed and analyzed in parallel. Samples and controls mixed with the fixative solution were processed immediately after sampling using the microbiology facility of the puy de Dôme’s atmospheric observatory. These were filtered on 0.22 μm porosity filters (MoBio 14880-50-WF), within a vertical laminar flow hood previously exposed to UV light for 15 minutes, the filters cut in halves with a sterile scalpel, and each half was finally transferred into bead-beating tubes of the MoBio Power Water kits for DNA or RNA extraction, and stored at -80°C until being further processed, within a week. Samples for routine analyses (cell counts and chemical analyses, see below) were collected during the course of sampling by temporarily exchanging the collection bottle containing the fixative solution for an empty, sterile, glass bottle, until enough volume (~10–15 mL) was collected (~30 min).

### 2.2. Cell counts and chemical analyses

Cloud water samples collected in the absence of fixative solution were used for chemical and microbiological characterization. Ion analysis was realized within a month by ion chromatography on Dionex DX320 for anions (column AS11, eluant KOH) and Dionex ICS1500 for cations (column CS16, eluant hydroxymethanesulfonate) on samples kept at -25°C, similarly as in [41]. Cell counts were performed by flow cytometry (BD FacsCalibur, Becton Dickinson, Franklin Lakes, NJ) on 450 μL triplicates added with 50 μL 5% glutaraldehyde (0.5% final concentration; Sigma-Aldrich G7651) stored for < 1week at 4°C. For analysis, samples were mixed with 1 vol. of 0.02 μm filtered Tris-EDTA pH 8.0 (40 mM Tris-Base, 1 mM EDTA, acetic acid to pH 8.0) and stained with SYBRGreen I (Molecular Probes Inc., Eugene, OR) from a 100X solution. Counts were performed for 3 minutes or 100,000 events at a flow rate of ~80 μL min^-1^ (precisely further determined by weighting). Prokaryotes and eukaryotes were distinguished from background particles based on fluorescence and side scattering light intensities (λ_exc_ = 488nm; λ_em_ = 530nm).

### 2.3. Meteorological data and backward trajectory plots

Meteorological variables were monitored continuously by the atmospheric observatory of the puy de Dôme’s summit at 5 min intervals. Details on the instrumentation can be found at http://wwwobs.univ-bpclermont.fr/SO/mesures/instru.php. Twenty-four hours backward trajectory plots of the air masses sampled were computed for the puy de Dôme’s site (45.772 N, 2.9655 E; 1465 m above sea level) using the NOAA HYSPLIT trajectory model (HYbrid Single-Particle Lagrangian Integrated Trajectory; [42] using GDAS (1degree) meteorological data archive and default parameters for this site.

### 2.4. Nucleic acids extraction, amplification and sequencing

DNA and RNA were extracted separately from dedicated filter halves using MoBio PowerWater isolation kits for DNA and for RNA, respectively, following manufacturer’s recommendations and including a DNase treatment step on RNA extracts. The absence (RNA fractions) or presence (DNA fractions) of DNA in the extracts was verified by PCR targeting the 16S rRNA gene of bacteria using the universal primers 1492r and 27f and similar PCR conditions as in [40]. From RNA extracts, cDNA were obtained using Superscript VILO cDNA synthesis kit (Invitrogen). Ribosomal RNA and RNA genes were then amplified and barcoded by PCR from DNA extracts and cDNA products using primer couples targeting either the V4 region of the 16S subunit of prokaryotes (primers 515F and 806R [43], or the V7 region of the 18S subunit of eukaryotes (primers 960F-1200R [44]; S1 Table). The “Marine” cloud was not processed for RNA due to issues related to storage of the corresponding extract. PCR were performed in total volumes of 30 μL, containing 3 μL of 10X NH_4_ reaction buffer, and final concentrations of 2 mM MgCl_2_, 0.75 units of of Eurobio Taq II DNA polymerase (Eurobio, 5U/μL), 0.2 mM each dNTP, 0.5 mg mL^-1^ BSA, and 0.2 μM of each primer. The amplification conditions consisted of an initial denaturation at 94°C for 5 min followed by 30 cycles of 1 min. at 94°C, 45 s. at 58°C (16S) or at 55°C (18S) and 45 s. at 72°C, ended by a final elongation step of 7 min. at 72°C. Amplicons length was verified by agarose gel electrophoresis then purified using MinElute Gel Extraction kits (Qiagen) before quantification by fluorescence using Quant-it PicoGreen (Molecular Probes Inc., Eugene, OR). Finally, an equimolar pool of 14 PCR products was prepared (total amount of 510 ng of DNA (17 ng DNA μL^-1^ in 30 μL molecular biology grade H_2_O). Further sample processing and sequencing was realized by Genoscreen (Lille, France). Briefly, DNA libraries were generated by adaptator ligation (section “Perform End Repair and Size Selection”, Illumina reagent kit V3), and controlled on Agilent High Sensitivity microarray. Sequencing (2×300 bp paired-end on Illumina MiSeq platform) yielded a total of 43 763 524 reads (13 129 Mb), 75.7% of which had a quality score Q30.

### 2.5. Sequence processing

A total of ~11.7 million reads were obtained from MiSeq sequencing. Prokaryotes, including Bacteria and Archaea, contributed ~6.6 millions reads in DNA and ~1.5 million reads in RNA (abbreviated into 16SDNA and 16SRNA, respectively), and eukaryotes ~2.5 millions reads in DNA and ~1.1 million reads in RNA (18SDNA and 18SRNA, respectively). These were assembled with the vsearch tool (https://github.com/torognes/vsearch) and cleaning procedures consisted in the elimination of sequences < 200 bp, presenting a mismatch in the forward or reverse primer, having ambiguous bases “N”, PHRED quality score < 25. The putative chimaeras were detected by vsearch. The remaining rRNA 16S (prokaryotes) and 18S (eukaryotes) sequences were clustered into “molecular species” (Operational Taxonomy Units, OTUs) at a 97% and 95% similarity threshold (OTU_0.03_ and OTU_0.05_, respectively), according to [45] and [46] with vsearch (option cluster_small sorted by length). The representative sequence for each OTU was inserted into phylogenetic trees for taxonomic annotation. The seed OTUs were finally affiliated by similarity and phylogeny from reference sequences extracted from the SSURef SILVA database [47], according to the following criteria: length > 1 200 bp, quality score >75% and a pintail value > 50. After comparing the OTUs with the reference sequences using a similarity approach (vsearch tool), trees including OTUs with their closest references were built with FastTree [48]. The different taxonomic affiliations obtained were checked for inconsistency. This process was implemented using the pipeline PANAM (Phylogenetic Analysis of Next-generation AMplicons https://github.com/panammeb/) and is described in more detail in [49,50].

The resulting OTUs were subjected to additional conservative filtering intended to remove potential sequencing artefacts (OTUs represented by less than 3 reads), contaminants (OTUs detected in the control samples) and phantom OTUs (OTUs detected in RNA and not in the DNA fraction of the corresponding sample), totals of 761 729 and 140 645 reads and 48 202 and 37 504 reads remained in the DNA and RNA fractions for prokaryotes and eukaryotes, respectively. The corresponding sequence files were deposited to NCBI’s Sequence Read Archive (SRA BioProject ID PRJNA380262). Data were and normalized (proportions) rather than rarefied to prevent loss of information and possible resulting biases [51]. Results obtained on datasets rarefied at different depth are summarized in S8 Fig for allowing comparison with other studies.

### 2.6. Data analyses

Data analyses were performed using the R environment version 3.2.2 [52], implemented with the *Phyloseq* package (version 1.18.1; [53]) for calculating Shannon-Wiener indexes and Abundance-based Coverage Estimators (ACE); *Phyloseq* was also employed for rarefying the datasets to depths similar as data found in the literature for richness comparison. Gini’s coefficient was calculated using the *ineq* package (version 0.2.13; [54]). Rarefaction curves were plotted using the ggplot2 package (version 2.2.0; [55]) from community analyses made with the *vegan* package (version 2.4.1; [56]); Venn diagrams were made using Venny 2.1.0 [57].

## 3. Results

### 3.1. Samples characteristics

The basic biological, chemical and meteorological features of the cloud water samples investigated are shown in Table 1; these were usual for clouds collected at the Puy de Dôme Mountain’s atmospheric observatory [40,58]. Based on geographical origin (S1 Fig), pH, and major ions composition, when available, these were classified into “Polluted”, “Continental” or “Marine” type events (S2 Fig)[58]. These categories comprise 9%, 26% and 52% of the clouds observed at puy de Dôme, respectively [58]. Total cell concentration was within the range typically observed in cloud water at this sampling site, with (2.05 to 9.49) ×10^3^ Bacteria and Archaea mL^-1^ and (0.4 to 8.7) ×10^2^ eukaryotic cells mL^-1^, equivalent to (0.4 to 2.5) ×10^3^ and 8 to cells 270 cells m^-3^ of cloudy air, respectively, depending on the sample (Table 1). Bacteria largely dominated the community, both in abundance (cell counts and read number) and richness (OTUs number): they represented ~90% of the DNA reads while eukaryotes contributed ~8%.and Archaea ~2%.

**Table 1:** Main characteristics of the samples.

				Biological data					Meteorological data								Chemical data						
Sample ID	Local sampling starting date and time	Local sampling ending date and time	Cloud type*	Sample volume filtered and used for sequencing (mL) [equivalent air volume (m^3^)]	Total prokaryotic cells concentration (mL^-1^)		Total eukaryotic cells concentration (mL^-1^)		T (°C)		Wind speed (m s^-1^)		Wind direction (°/N)		LWC^b^ (g m^-3^)			Dissolved ions concentrations (μM)					
					Mean	SE^a^	Mean	SE^a^	Mean	SE^c^	Mean	SE^c^	Mean	SE^c^	Mean	SE^c^	pH	Na^+^	SE^a^	Cl^-^	SE^a^	NO_3_^-^	SE^a^
**CLOUD 1**	10/11/13 10:36 AM	10/11/13 1:25 PM	Polluted	94 [304 m^3^ of cloudy air]	8.23×10^3^	± 1.25×10^3^	8.67×10^2^	± 3.15×10^2^	-1.2	± 0.2	ND*	-	ND*	-	0.16	± 0.06	4.2	ND	-	ND	-	ND	-
**CLOUD 2**	10/14/13 10:00 PM	10/15/13 11:10 AM	Continental	350 [1422 m^3^ of cloudy air]	9.49×10^3^	± 2.87×10^3^	2.83×10^2^	± 1.05×10^2^	6.5	± 0.3	12.1	± 1.5	218	± 9	0.31	± 0.05	4.7	79.0	± 6.5	15.5	± 2.8	38.5	± 0.8
**CLOUD 3**	11/5/13 2:45 PM	11/6/13 1:30 PM	Marine	420 [2457 m^3^ of cloudy air]	2.05×10^3^	± 1.39×10^3^	4.42×10^1^	± 0.21×10^2^	6.8	± 0.8	18.3	± 3.4	337	± 4	0.40	± 0.07	5.6	188.5	± 12.6	0.2	± 0.1	1.13	± 0.7

ND: Not determined.

*According to the classification of clouds sampled at the puy de Dôme as established by [58] (see S2 Fig), based on pH, ion content and backtrajectory plots (shown in S1 Fig).

^a^Standard error of 3 replicate measurements.

^b^Liquid water content.

^c^Standard error of averages of meteorological variables collected every 5 minutes during sampling.

### 3.2. The cloud water microbiota: An extremely rich and imbalanced community

A total of 28,143 OTUs were detected in prokaryotes (28,058 in Bacteria and 85 in Archaea) and 2,612 OTUs in eukaryotes. Each sample contained a fraction of the total richness, with ca. 7,800 to 20,500 OTUs_0.03_ in prokaryotes and ca. 1,900 to 2,100 OTUs_0.03_ in eukaryotes (Table 2). Inflexions in the rarefaction plots of the different sets of sequences (S3 Fig) indicated that the actual microbial communities targeted were well captured (coverage of 72% to 98%). The corresponding abundance-based coverage estimators (ACE) specified the presence of between ~10,800 and ~21,000 prokaryotic OTUs_0.03_ per sample and ~2,400 eukaryotic OTUs_0.05_ (Table 2). The results concerning specifically the composition of prokaryote, eukaryote, then active communities are presented below.

**Table 2:** Prokaryotic and eukaryotic communities’ richness and distribution.

	Polluted type cloud		Continental type cloud		Marine type cloud
	DNA	*RNA*	DNA	*RNA*	DNA
**Bacteria and Archaea**					
**Processed read number**	496,197	*59*,*449*	115,515	*81*,*196*	150,017
Total species richness*					
Observed	20,432	*3*,*428*	7,793	*5*,*622*	8,970
Estimated (ACE)	20,954	*4*,*710*	10,802	*6*,*468*	11,148
**Community distribution**					
Shannon's H	9.1	*6*.*3*	7.2	*7*.*3*	7.4
Gini coefficient	0.74	*0*.*98*	0.95	*0*.*95*	0.94
**Eukaryotes**					
**Processed read number**	18,562	*12*,*831*	13,978	*24*,*673*	15,662
Total species richness**					
Observed	2,061	*1*,*527*	1,901	*1*,*692*	1,877
Estimated (ACE)	2,461	*1*,*845*	2,400	*1*,*834*	2,439
**Community distribution**				* *	
Shannon's H	6.58	*6*.*28*	6.48	*6*.*32*	6.26
Gini coefficient	0.75	*0*.*81*	0.77	*0*.*81*	0.79

*OTUs clustered at 97% sequence similarity.

**OTUs clustered at 95% sequence similarity.

#### 3.2.1 Prokaryote community

**Fig 1:**
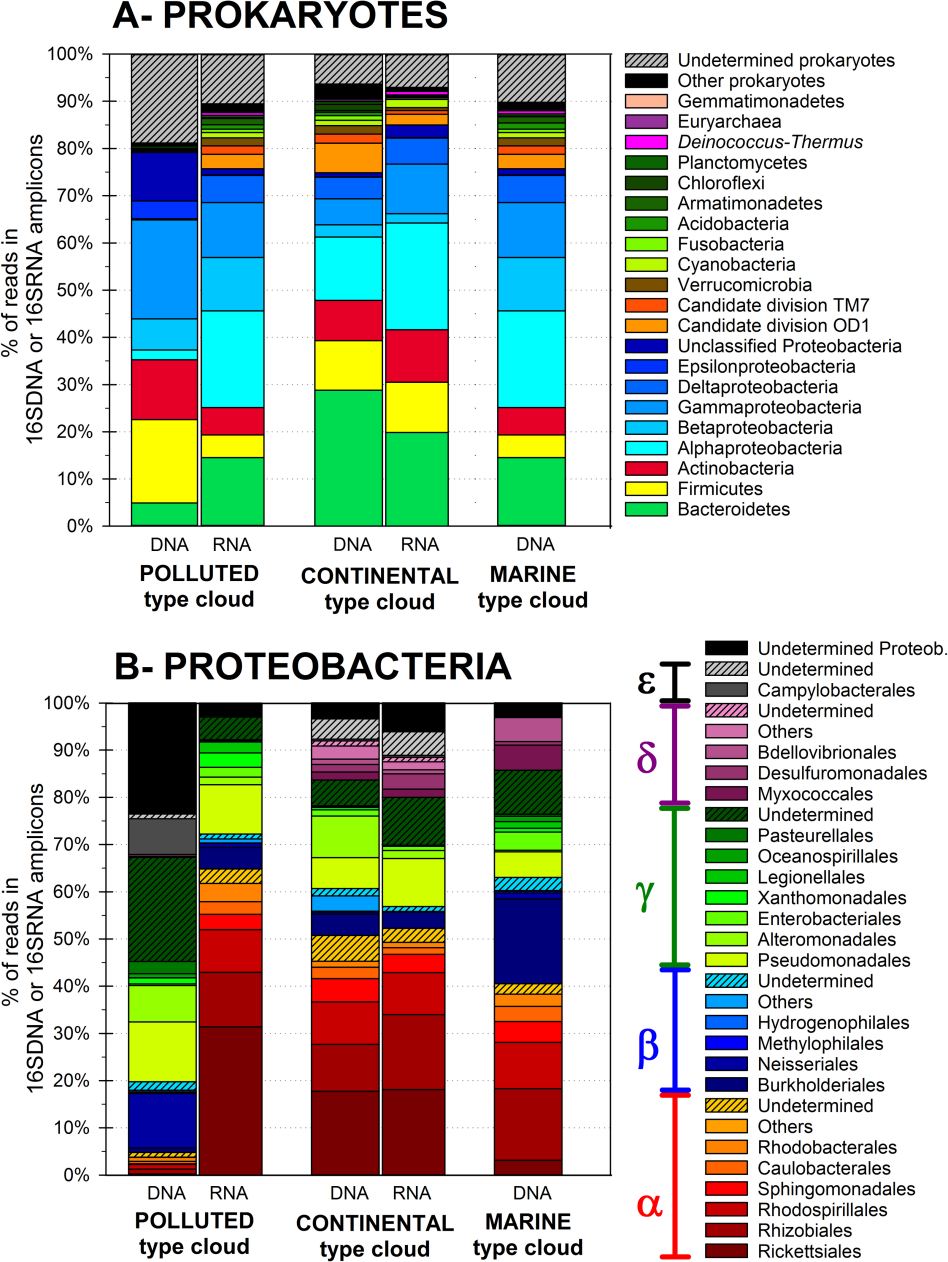
**Prokaryotic total (DNA fraction) and active (RNA fraction) community composition in the cloud water samples** at the phylum level (**A**),and relative distributions of Proteobacteria orders (**B**).

In total, 30 different phyla were detected (of which 1 in Archaea), distributed over 60 classes, 113 orders, 190 families and 286 genera. A complete list of the abundance and taxonomic affiliation of prokaryotic OTUs is presented in S1 File. In all samples, the community was largely dominated by 4 bacterial phyla, which represented 75% to 79% of the reads in DNA datasets and 85 to 88% in RNA (Fig 1A): Proteobacteria (28 to 51% of the DNA reads, in particular the Gamma- (6–21%), Alpha- (2–21%) and Beta- (3–12%) classes; Fig 1B), Bacteroidetes (5–30%), Firmicutes (5–18%) and Actinobacteria (6–13%). These phyla are almost systematically reported dominant in outdoor airborne bacterial communities studies (e.g. [3,6,8,59–62]).

A total of 1,593 OTUs distributed over 103 genera were common to all samples (S4A Fig). These represented 64% to 96% of the reads identified down to this taxonomic level, and 15%-31% of the total 16SDNA reads in each sample. Their relative contribution to the whole community structure in the different samples is shown as a heat-map in S5A Fig. Dominant genera comprised notably: *Rickettsia*, *Sphingomonas*, *Methylobacterium* and *Acidiphilium* in Alpha-Proteobacteria; *Pseudomonas*, *Enhydrobacter*, *Moraxella* and *Psychrobacter* in Gamma-Proteobacteria; *Capnocytophaga* in Bacteroidetes; *Corynebacterium*, *Arthrobacter* and *Streptomyces* in Actinobacteria; *Dolosigranulum*, *Clostridium* in Firmicutes.

The samples had clear distinct patterns: ~70% of the total OTU richness observed in prokaryotes was contributed by the “Polluted” type sample, with most of them being characteristic, *i*.*e*. exclusive of this sample (15,152 OTUs representing 72% of the reads of this sample). Comparatively, other samples had 2,600 to 3,000 characteristic OTUs which represented 8–12% of the reads. The “Polluted” type cloud was characterized by relatively high abundance of *Dolosigranulum*, *Corynebacterium*, *Moraxella* and *Campylobacter* bacteria. The “Marine” type cloud was dominated by Proteobacteria affiliated with *Bdellovibrio*, *Pseudomonas*, *Methylobacterium*, *Sphingomonas* and *Rickettsia*; these were also well represented in the “Continental” type cloud, along with some Firmicutes and Actinobacteria (*Clostridium*, *Streptococcus* and *Corynebacterium*).

#### 3.2.2 Eukaryote community

**Fig 2:**
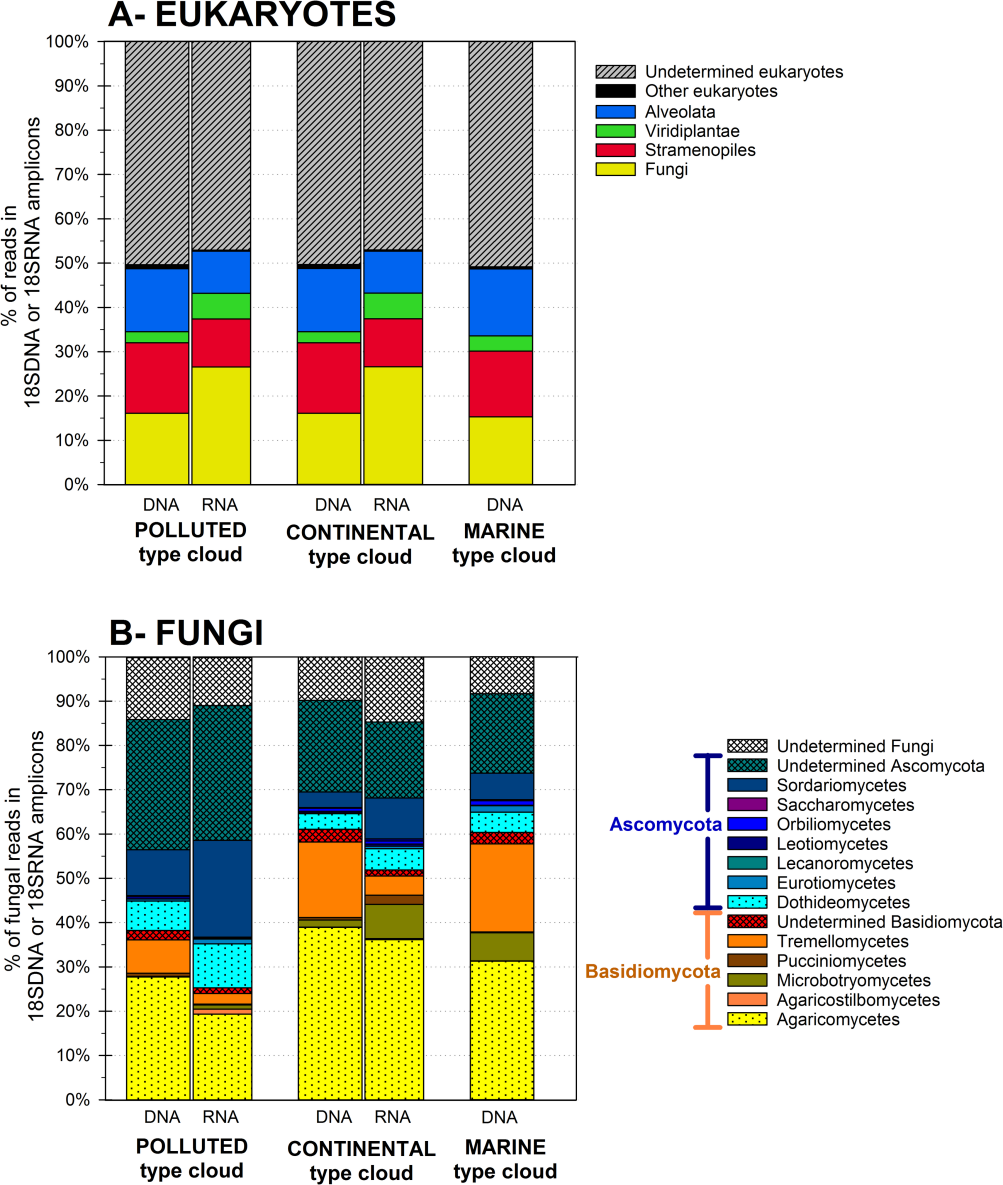
**Eukaryotic total (DNA fraction) and active (RNA fraction) community composition in the cloud water samples** at the kingdom level (**A**), and relative distributions of Fungal classes (**B**).

Eukaryotic OTUs were distributed over 12 phyla, with 66 orders identified. A complete list of the abundance and taxonomic affiliation of eukaryotic OTUs can be found in S2 File. A large proportion of reads (~50%) remained unaffiliated at the phylum level, both in DNA and RNA. The reads taxonomically identified in the DNA fraction were evenly distributed among Fungi, Stramenopiles and Alveolata (12% to 18%), while Viridiplantae represented ~3% (Fig 2A). Basidiomycota and Ascomycota largely dominated in Fungi (Fig 2B). By far, most identified Basidiomycota were members of the classes Agaromycetes (52% to 73%, with Polyporales and Agaricales the dominant families), Tremellomycetes (20% to 33%) and Microbotryomycetes (0.2% to 11%). In the phylum Ascomycota, among those identified to the class level, Sordariomycetes (12% to 22%) and Dothideomycetes (12% to 15%) dominated; other classes (Eurotiomycetes, Lecanoromycetes, Leotiomycetes, Orbiliomycetes and Saccharomycetes) represented < 5% of the reads.

A total of 1,209 eukaryotic OTUs were shared between the samples (S4B Fig). These were distributed over 39 orders, gathering 15.2% to 16.6% of the total reads of the samples and 91% to 97% of those identified at this taxonomic depth. Their relative abundance in the eukaryotic communities of each sample is shown as a heat-map in S5B Fig. Dominant shared orders in all samples included notably Bicoseocida, Polyporales, Charales and Euplotida.

### 3.3. Active community

The active community, as detected in RNA extracts, was a fraction of the total community. This included 26.4% of the total richness observed in prokaryotes (7,438 OTUs_0.03_) and 82% (2,612 OTUs_0.05_) in eukaryotes. The samples were globally less distinct in their RNA fraction than they were in their DNA fraction (S6 Fig). A total of 1,612 prokaryotic OTUs were shared between the RNA fractions of 2 samples analyzed. These were distributed over 97 identified genera gathering in total 32% to 34% of the 16SRNA reads, of which a few dominant genera contributed each around 1%: *Rickettsia*, *Spirosoma*, *Enhydrobacter*, *Corynebacterium*, *Acidiphilium*, *Sphingomonas*, *Pseudomonas* and *Methylobacterium*. In eukaryotes, most RNA reads (18% to 27%) were attributed to Fungi, whereas Stramenopiles and Alveolata each were represented by ~10%, and Viridiplantae by ~6%. A the order level, dominant Fungi included Magnaporthales and Pleosporales in Ascomycota, Polyporales and Sporidiobolales in Basidiomycota), SAR (Bicosoecida) and others such as Syndiniales, a group of dinoflagellates.

**Fig 3:**
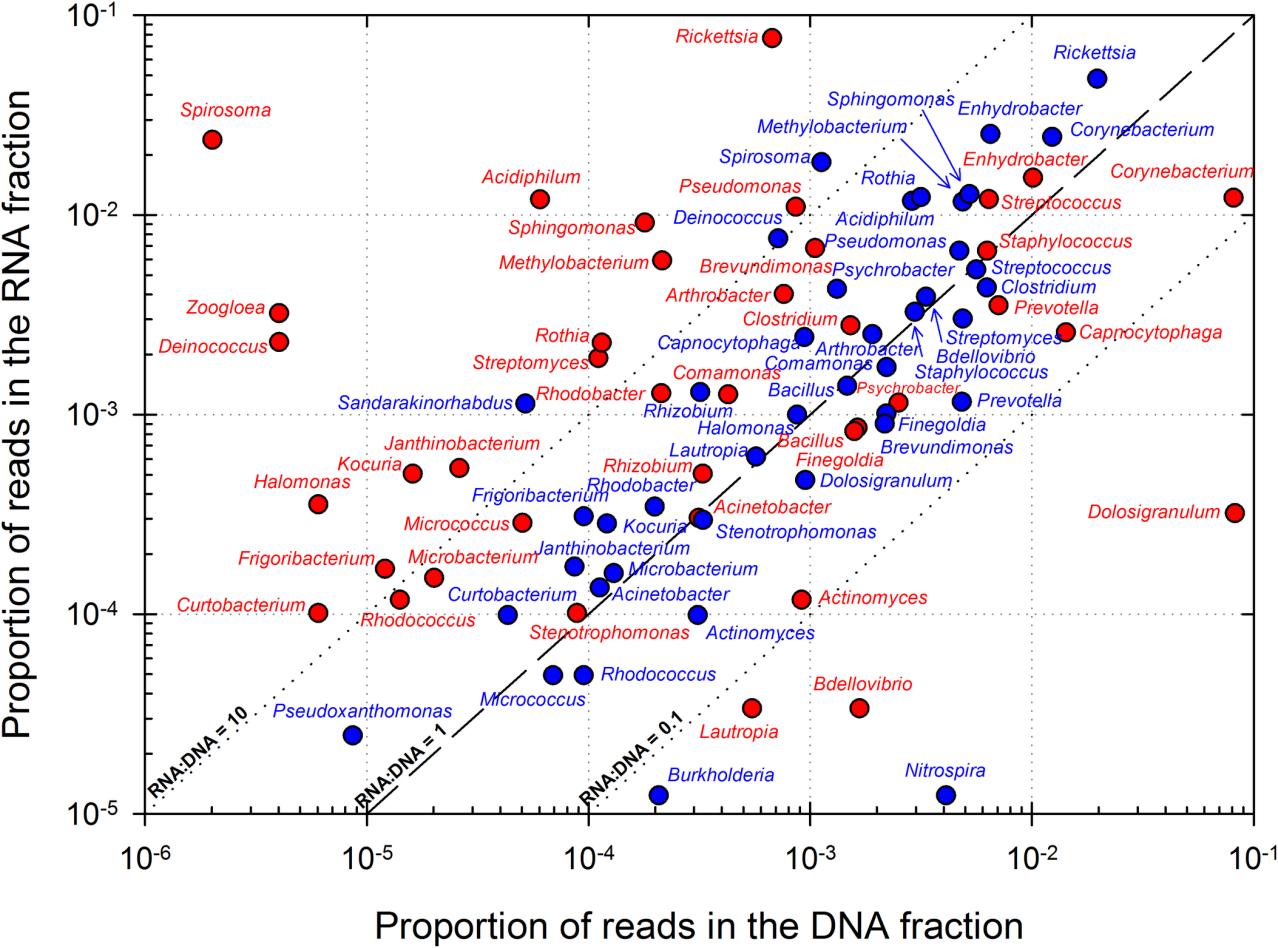
Representation of the major prokaryotic genera identified in DNA and RNA datasets. Dashed and dotted lines depict RNA:DNA ratios of 0.1, 1 and 10. The top 20 genera based on their average position rank over the 3 cloud samples are shown, as well as some selected for high representation in RNA datasets (43 distinct genera in total). POLL: “Polluted” type cloud; CONT: “Continental” type cloud.

**Fig 4:**
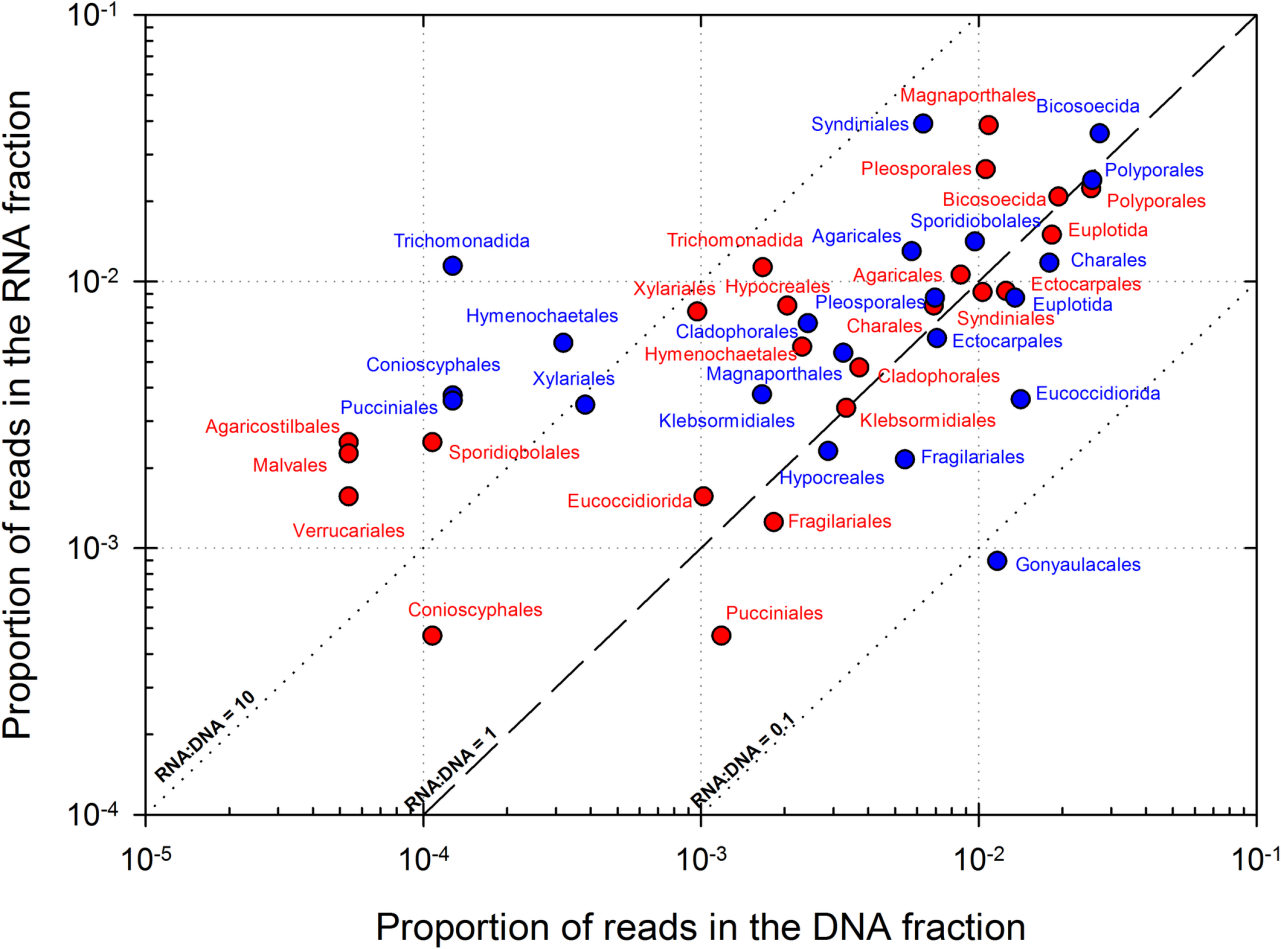
Representation of the major eukaryotic orders identified in DNA and RNA datasets. Dashed and dotted lines depict RNA:DNA ratios of 0.1, 1 and 10. The top 20 genera based on their average position rank over the 3 cloud samples are shown, as well as some selected for high representation in RNA datasets (24 distinct orders in total). POLL: “Polluted” type cloud; CONT: “Continental” type cloud.

Figs 3 and 4 compile overall most represented bacterial genera and eukaryotic orders, respectively, in corresponding DNA and RNA datasets. The relative abundance of RNA, respect to DNA, in an OTU (abbreviated into RNA:DNA ratio for clarity) is often used for estimating its relative level of metabolic activity, with higher ratios linked with potentially higher metabolic activity [63,64]. RNA:DNA ratio ranged between 0 and 210 in eukaryotes, and from 0 to, exceptionally, 11,760 in prokaryotes in an OTU affiliated to *Spirosoma* (Bacteroidetes). Low abundance groups tended to exhibit high ratios, in prokaryotes and in a lesser extent also in eukaryotes (see S7 Fig), as observed by others in atmospheric samples [65], but by far, most RNA:DNA ratios were between 0.1 and 10. Alpha- and Gamma-Proteobacteria clearly dominated in bacterial taxas with ratio > 1 (i.e. potentially metabolically active taxa). Notably *Rickettsia*, *Sphingomonas*, *Methylobacterium*, *Enhydrobacter*, *Pseudomonas*, and *Acidiphilium* genera were highly represented and were probably the most active taxas. In bacteria, these included notably *Spirosoma*, *Deinococcus* (Deinococcus-Thermus), *Janthinobacterium* (Beta-Proteobacteria), *Frigoribacterium* and *Curtobacterium* (Actinobacteria). Conversely, some bacteria were found abundant but exhibited very low or no activity based on RNA:DNA ratio. These comprised essentially Gram-positive bacteria: Actinobacteria (*Corynebacterium*, *Actinomyces*) and Firmicutes (*Dolosigranulum*, *Staphylococcus*), and also members of Proteobacteria (*Bdellovibrio*, *Burkholderia*), Bacteroidetes (*Capnocytophaga*) and others like *Nitrospira*. In eukaryotes the orders Magnaporthales, Syndiniales, Pleosporales, Polyporales, Bicosoecida and Sporidiobolales in particular were markedly abundant in both the DNA and RNA datasets.

## 4. Discussion

### 4.1. Clouds are extremely rich and diverse mosaics of multiple sources ecosystems

In this work, we aimed at drawing a picture of the structure of cloudborne microbial communities, including active groups and rare taxa. The detection of eventual environmental drivers such as meteorological variables to the microbial communities observed was beyond the scope of this study. Thus, we chose to orient our investigations toward large sample volumes, associated with deep sequencing. Consistently, species richness reaches here an unprecedented value in atmospheric samples, with ~11,000 to ~21,000 distinct OTUs estimated in prokaryotes and ~2,400 in eukaryotes. Such high richness are uncommon and in general rather reported in soils (e.g., [66,67]. In the atmosphere, although much less is known, it is often described as a highly diverse environment (e.g., [7,8,60,68–70]. The high richness observed in our samples can be related to the large sample volumes considered. It is clear that scale problems arise when estimating community richness in open ecological systems [71,72], especially in dynamic environments like the atmosphere where the biomass is low. Comparatively, DNA analyses were carried out in reference studies from 2.7 to 144 m^3^ of air at mid-altitude sites (ca. 1,500 to 3,000 m asl.; [35,65], and ~6 m^3^ of air in the free troposphere (i.e. ~3×10^4^ bacterial cells per sample; [10]). Volumes similar as in our study were notably used for assessing fungal [73] and prokaryotic diversity [4] in aerosols at global scale: up to 1,100 species of fungi and 2,900 species of prokaryotes per sample were observed. Recently [62] detected up to 1,910 species in cloud water volumes of 100 mL, on data rarefied to 9,100 sequences. A richness equivalent was observed in our study when rarefying data to a comparable depth (S8 Fig). Finally, bacterial species richness similar as our findings was reported from large rain samples (7–22 liters), with 13,083 OTUs_0.03_ [74], in [61].

The structure of the communities was investigated through ecological indexes (Table 2). Shannon’s H indexes ranging from 6.3 to 9.1 demonstrated extreme biodiversity, in a large part contributed to by the numerous rare species. Indeed, the communities, in particular prokaryotes, were highly uneven with a low proportion of abundant species and many rare, as shown by OTU rank-abundance plots (S3C and S3D Fig), Lorenz curves (S9 Fig) and corresponding Gini’s coefficients close to 1 (0 being a perfect equality in OTU abundance distribution and 1 being perfect inequality, *i*.*e*. a very contrasted abundance distribution between OTUs). Illustrating this, despite conservative sequence filtering, the 1% most abundant OTUs represented around ~20% of the reads in eukaryotes and ~35% in prokaryotes, respectively, and the top half OTUs more than 90%. This high unevenness suggests that the global functioning of the community is fragile (not robust), sensitive to stress [75], and so, likely to be variable in space and time. If an abundant group was to be lost from the community, i.e. a group that is likely to contribute significantly to the structure and global functioning of the system, there would be a high probability to lose or reduce also the functions associated with it. This ecological theory, that functional stability implies even structure, derives from established ecosystems and it is applied here for apprehending the functioning of cloud’s microbial communities in the frame of clouds as microbial habitats hypothesis; it is possible though that this is not applicable to environments acting mainly as transport areas, where microbial establishment is by essence not possible, like clouds.

Any microbe inhabiting a surface environment has a probability to get aerosolized, though more or less promptly depending on its physiological characteristics (e.g.,[76] and other environmental variables linked with its habitat, like exposure to mechanical disturbances by wind or rainfall for instance [77–79]. The community observed likely resulted from the mixing of microbial inputs from myriads of different sources, albeit not confidently quantifiable here. The high unevenness observed could suggest by itself that there is a marked influence of some specific environments over others; this assumes temporal stability on sources and equivalent strengths among sources and microorganisms, and this is probably not the case (e.g., [67,80,81]. Rather, the variability between the samples indicated that the sources themselves are large and rich, so a variety of possible children communities can emerge from it [82].

As needles in a haystack of complex communities, the presence of more or less specific tracers can inform about emission sources. It is widely observed, and our samples are no exception, that airborne microorganisms outdoors most likely originate from soil, vegetation, surface waters and animals among natural sources; Humans and activities such as composting can also create bioaerosols in high number (e.g., [35,76,79,83,84]). Proteobacteria and Bacteroidetes dominated the communities observed, with also a good representation of Actinobacteria and Firmicutes, as usually in airborne microbial communities [6,8,10,59,60,85]. Prokaryotic endosymbionts or parasites of eukaryotes (*Rickettsia*, *Wolbachia*)[86,87] were present in high proportion. To our knowledge, this is the first report of such abundance of these organisms in atmospheric samples. Their presence is not surprising as they probably originated from the numerous plant, insect, arthropod and other animal fragments contained among aerosols [88]. *Rickettsia* are ultra-small bacteria thought to be at the origin of mitochondria in eukaryotes (endosymbiotic theory) [89]. The abundance of Pseudomonads and Sphingomonads attested of important inputs from vegetation in all samples (e.g., [90,91]), whereas others like *Streptomyces* rather refer to soils. These apart, *Bdellovibrio*, a genus of Delta-Proteobacteria found in water environments, dominated in the “Marine” type cloud was, and taxa generally affiliated with soils, decomposing organic material, animals, and humans, like *Dolosigranulum*, *Corynebacterium*, *Moraxella*, *Campylobacter* and *Capnocytophaga* (e.g., [92]) were abundant in the “Polluted” type cloud. Wei et al. studied polluted and non-polluted fog events in China and also observed a prevalence of potential Human pathogens in the polluted air masses [61].

In eukaryotes, Basidiomycota tended to dominate over Ascomycota, as a result from continental inputs [11,73,93]. The relative dominance of Basidiomycota over Ascomycota in the air was revealed recently by culture-independent methods [11,93]. More precisely, Basidiomycota tend to dominate in continental air masses, whereas Ascomycota prevail in marine air masses [73].

Despite sampling site’s remoteness from ocean, samples kept a marine biological signature detectable on the taxonomic affiliation of some abundant groups of microorganisms, as also observed for chemical composition: marine or water-related taxa, in prokaryotes (*Bdellovibrio*, Delta-Proteobacteria) and in eukaryotes [green, brown and red algae (Charales, Ectocarpales, Hildenbrandiales, Fucales, Cladophorales), diatoms (Fragilariales, Thalassiosirales, Hemiaulales), fungi and protozoans affiliated with water environments (Syndiniales, Saprolegniales, Conioscyphales)]. Saprophytic fungi affiliated with vegetation, soils and decomposing litters were also particularly abundant: Polyporales, Sporidiobolales, Agaricales, Pleosporales, and others.

Although it is not statistically verifiable, we observed that the prokaryotic community, and in a lesser extent the eukaryotic community, were richer (ACE estimator), more diverse (Shannon’s index), and less uneven (Gini’s coefficient) in the “Polluted” type cloud than in non-polluted “Continental” or “Marine” type clouds (Table 2). A relationship between Human activities and microbial communities structure in clouds was reported in China [61], with higher diversity in non-polluted clouds. Another study rather pointed out an impact of day and night on the composition of bacterial communities in clouds; a higher representation of Alpha-Proteobacteria during the night, notably, was reported [62], but the reasons for such trend are not clear.

### 4.2. Clouds are environments open to all, but where only some can thrive: active groups

Among the high diversity of cloud microbial communities, some were capable of maintaining metabolic activity in cloud despite probable stressful conditions. According to criteria of abundance in both DNA and RNA fractions, RNA:DNA ratio (Fig 3), frequency of recovery in cultures in earlier studies [40], and other hints from previous reports [10,65], and at the exception of eukaryotic endosymbionts (*Rickettsia*), these probable main bacterial “inhabitants” of clouds can be named: Alpha- and Gamma-Proteobacteria, in particular *Sphingomonas* (order Sphingomonadales), *Methylobacterium* (Rhizobiales), *Acidiphilium* (Rhodospirillales), *Pseudomonas* (Pseudomonadales), *Comamonas* (Burkholderiales) and, to a lesser extent, *Enhydrobacter* and *Psychrobacter* (Pseudomonadales). Among more discrete genera, *Curtobacterium*, *Deinococcus*, *Spirosoma*, *Rhizobium* and *Janthinobacterium* notably can also be cited here, along with, in other phyla, *Arthrobacter*, *Staphylococcus*. All these have physiological properties compatible with their maintenance in the high atmosphere and clouds, and they probably interact with their cloud water environment with potential impacts on chemistry. Many of these are epiphytic taxa commonly recovered viable from air and clouds [8,9,17,29,84,94–96]. On the other hand, tracers of polluted air masses could reside amongst the most abundant species in DNA, like *Dolosigranulum* or *Capnocytophaga*.

Many of the microorganisms identified relate to vegetation: epiphytic, parasitic and endosymbiontes. Plant leaves, like clouds, are subjected to frequent temperature and humidity shifts, high levels of UV light, etc. It is possible that these bacteria acquired physiological traits compatible with survival in clouds from this lifestyle. *Pseudomonas* and *Sphingomonas* species are versatile bacteria abundant in the environment, particularly on vegetation (e.g., [80]). *Pseudomonas* are among the bacteria the most frequently recovered by culture (i.e. viable) from clouds and atmospheric samples [17,27,40], where their presence is particularly interesting for many reasons: plant pathogenicity and epidemiology, degradation of organic compounds in clouds [97,98]; production of siderophores and interactions with iron and radical chemistry [99]; production and release of surfactants, which could facilitate the formation of cloud water droplets [21,68]; ice nucleation, which in clouds can trigger precipitation (hypothesized as “bioprecipitation”) (e.g.[96]). *Sphingomonas* are pigmented oligotrophic bacteria, frequently described as psychrotolerant bacteria recovered from polar environments and air samples [36,100,101]. Many of them are studied for their intrinsic resistance to numerous antibiotics [102], and for their capacity to degrade xenobiotics [103], alike *Comamonas* [104]. *Methylobacterium* are methylotrophic bacteria, i.e. they can develop on one-carbon compounds such as methanol, formaldehyde or formate [105,106], which are abundant in the atmosphere and in cloud water [41,98]. Some species can use compounds shown to be responsible for ozone depletion in the stratosphere, such as chloromethane [107]. The presence and potential activity of methanotrophic bacteria in the air was shown previously [32]. *Acidiphilium* have high capacities of interaction with iron [108,109] and so is a good candidate for interfering with cloud water oxidant capacity [33]; it remains yet rarely isolated by culture. *Enhydrobacter* have gas-vacuole helping floatation in aquatic environments [110], and it is possible that this favored its aerosolization from waters bodies (e.g., 104). Finally, *Deinococcus* and *Spirosoma* are known for their high resistance to DNA-damages such as those caused by UV light [111,112], so their presence among the common core of the community is no surprise. *Spirosoma* species have been described from Arctic and Mountain regions [112,113].

In eukaryotes, endosymbiontes and parasites flagellate protists (Syndiniales and Bicosoecida) dominated in DNA, but active groups included mainly plant pathogens and saprophytic fungi from terrestrial or aquatic origins known for aerial dispersion [114–116] in Ascomycota (Pleosporales, Magnaporthales, Xylariales and Conioscyphales), and Basidiomycota (Pucciniales, Hymenochaeales and Sporodiobolales) (Fig 4). Ascomycota were previously reported dominant in the active fraction of airborne fungi [93], and in Basidiomycota, Sporodiobolales includes yeasts frequently isolated from cloud water samples at the same site, *Rhodotorula* and *Sporobolomyces* [40], However, if RNA:DNA ratio gives hints about potentially active eukaryotes, ribosome gene number is intrinsically more variable than it is in prokaryotes [117]; so estimating their actual relative activity in cloud water will necessitate more investigations.

Our investigations revealed an incredible richness in the atmosphere, originating from a variety of different sources and meeting in clouds. High inequities suggested high sensibility to perturbations, including potentially stress caused by Human activities. Frequent species probably composed most of the biomass, but the vast majority of the diversity was contributed by rare species. This feature, common in the environment (e.g., [118], funded the “everything is everywhere” concept (e.g., [119]. There is no “global atmosphere” with a specific community structure and functioning, but rather a multitude of different regional to local atmospheres distributed over the globe, as moving airborne imprints of surface ecosystems. On top of this, some atmospheric corridors connecting distant regions together and defining some extent of bio-geographical distribution of microorganisms on the planet have been identified [5,73,118].

Airborne communities are sorts of blurred airborne imprints of surface ecosystems gathering and overlapping with each other in clouds A set of microorganisms able to maintain metabolic activity in clouds was identified among complex communities. In previous studies, many of these active taxa were frequently recovered by culture from cloud water samples [29,40]. These represent the microorganisms the most prone to interfere with their cloud chemical environment. They are also potential competitors brought to surface receptacle ecosystems by atmospheric deposition, and the early colonizers of emerging environments. Their identification certainly helps understanding the atmosphere as a habitat; it will also allow focusing researches for evaluating microbial impact on cloud physical and chemical processes, but their actual functioning, the “*what do they do*?” question remains to be answered.

## Supporting information

S1 File 16S rRNA and 16S rRNA gene amplicons taxonomic affiliations and abundances in the datasets. (XLSX)

S2 File 18S rRNA and 18S rRNA gene amplicons taxonomic affiliations and abundances in the datasets. (XLSX)

S1 Table Oligonucleotide sequences of primers and indexes used for barcoded PCR amplification of prokaryotic and eukaryotic ribosomal genes. (DOCX)

S1 Fig Twenty-four hour backward trajectory plots of the air masses corresponding to the cloud event sampled. (TIF)

S2 Fig Principal component analysis used for categorizing clouds at this sampling site based on chemical composition, implemented from [58]. (TIF)

S3 Fig Rarefaction curves (**A and B**) and rank-abundance plots (**C and D**) of the different set of amplicons.
(TIF)

S4 Fig Venn diagrams depicting similarities and singularities of the 3 samples at the OTU_0.03_ level for prokaryotes (**A**) and OTU_0.05_ for eukaryotes (**B**).
(TIF)

S5 Fig Relative abundance of shared prokaryotic genera **(A)** and eukaryotic orders **(B)**.
(TIF)

S6 Fig Bray-Curtis similarity matrices between the different sets of sequence of prokaryotes (**A**) and eukaryotes (**B**).
(TIF)

S7 Fig Relative OTU representation in extracts of RNA respect to DNA in corresponding datasets, showing a trend of higher RNA.DNA ratios in rare OTUs. (TIF)

S8 Fig Observed OTUs, richness estimators and Shannon’s indexes calculated at different rarefactions depths. (TIF)

S9 Fig Lorentz curves of the different sets of sequences. (TIF)
