# Supplementary material for: Active microorganisms thrive among extremely diverse communities in cloud water
Source: PLoS One. 2017 Aug 8;12(8):e0182869. doi: 10.1371/journal.pone.0182869 (PMC5549752; doi:10.1371/journal.pone.0182869)
Supplement: S1 Table — (DOCX) [file pone.0182869.s003.docx]

**S1 Table:** Oligonucleotide sequences of primers and indexes used for barcoded PCR amplification of prokaryotic and eukaryotic ribosomal genes.

| **Target genome region** | **Primer or tag name** | **Sequence* (5'-3')** |
| --- | --- | --- |
| V4 region of the 16S rRNA gene of prokaryotes | 515F^a^ | GTGYCAGCMGCCGCGGTA |
|  | 806R^b^ | GGACTACHVGGGTWTCTAAT |
| V7 region of the 18S rRNA gene of eukaryotes | 960F^c^ | GGCTTAATTTGACTCAACRCG |
|  | 1200R^c^ | GGGCATCACAGACCTGTTAT |
| Indexes (5'-3') | F1 | ACACACAC |
|  | F2 | ACAGCACA |
|  | F3 | GTGTACAT |
|  | F4 | TATGTCAG |
|  | F6 | TACTATAC |
|  | F7 | ACTAGATC |
|  | F9 | CGCTCTCG |
|  | F10 | GTCGTAGA |
|  | F12 | GACTGATG |
|  | F13 | AGACTATG |
| * IUPAC nomenclature |  |  |
| a: from [1] |  |  |
| b: from [2] |  |  |
| c: from [3] |  |  |

1. Caporaso JG, Lauber CL, Walters WA, Berg-Lyons D, Huntley J, Fierer N, et al. Ultra-high-throughput microbial community analysis on the Illumina HiSeq and MiSeq platforms. ISME J. 2012;6: 1621–1624. doi:10.1038/ismej.2012.8

2. Pinto AJ, Raskin L. PCR Biases Distort Bacterial and Archaeal Community Structure in Pyrosequencing Datasets. PLoS One. 2012;7. doi:10.1371/journal.pone.0043093

3. Gast RJ, Dennett MR, Caron DA. Characterization of Protistan Assemblages in the Ross Sea, Antarctica, by Denaturing Gradient Gel Electrophoresis. Appl Environ Microbiol. 2004;70: 2028–2037. doi:10.1128/AEM.70.4.2028-2037.2004
