# Supplementary figures and images for: Active microorganisms thrive among extremely diverse communities in cloud water
Source: PLoS One. 2017 Aug 8;12(8):e0182869. doi: 10.1371/journal.pone.0182869 (PMC5549752; doi:10.1371/journal.pone.0182869)

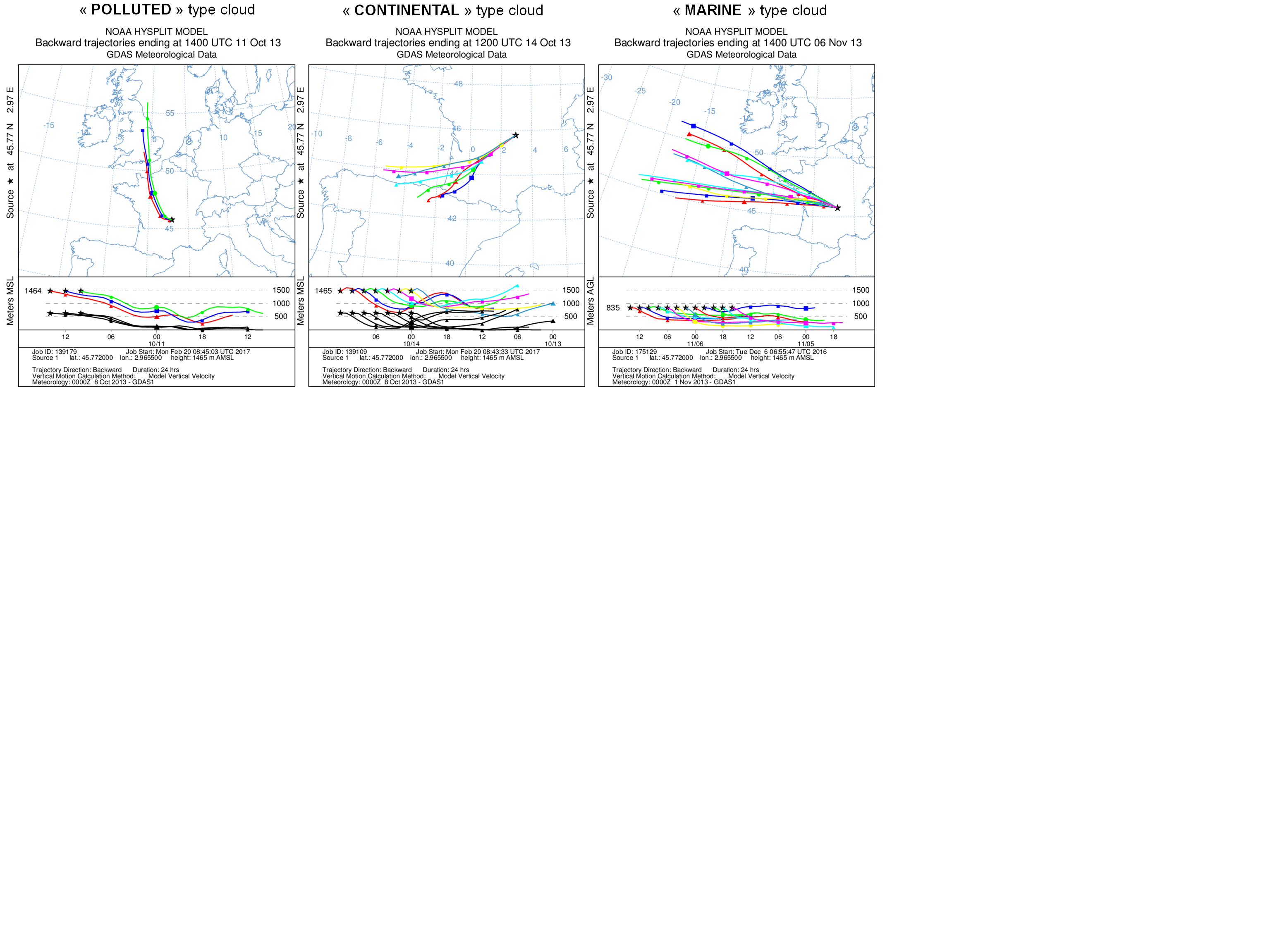

Supplement: S1 Fig — (TIF) [file pone.0182869.s004.tif]

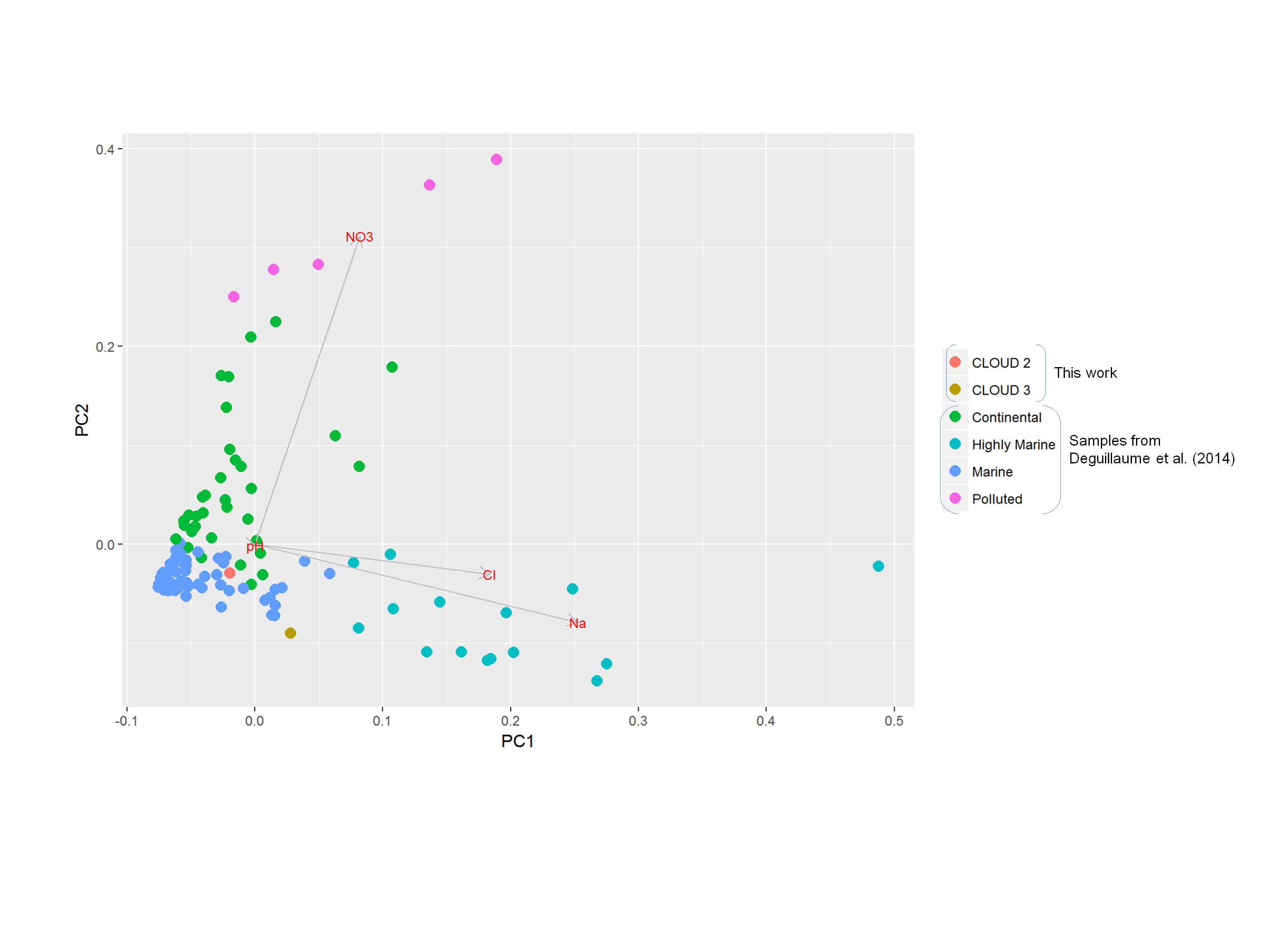

Supplement: S2 Fig — (TIF) [file pone.0182869.s005.tif]

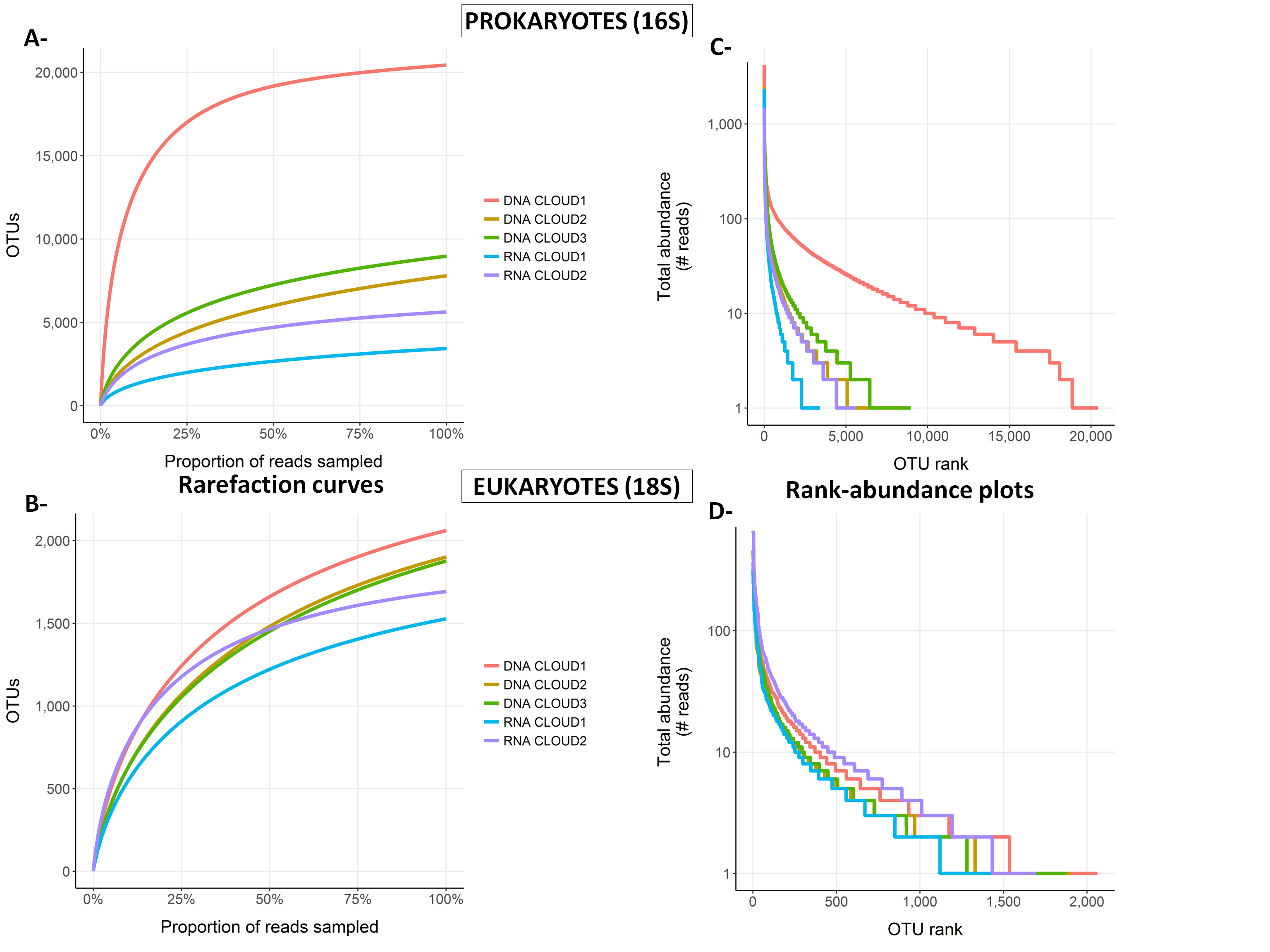

Supplement: S3 Fig — Rarefaction curves (A and B) and rank-abundance plots (C and D) of the different set of amplicons. (TIF) [file pone.0182869.s006.tif]

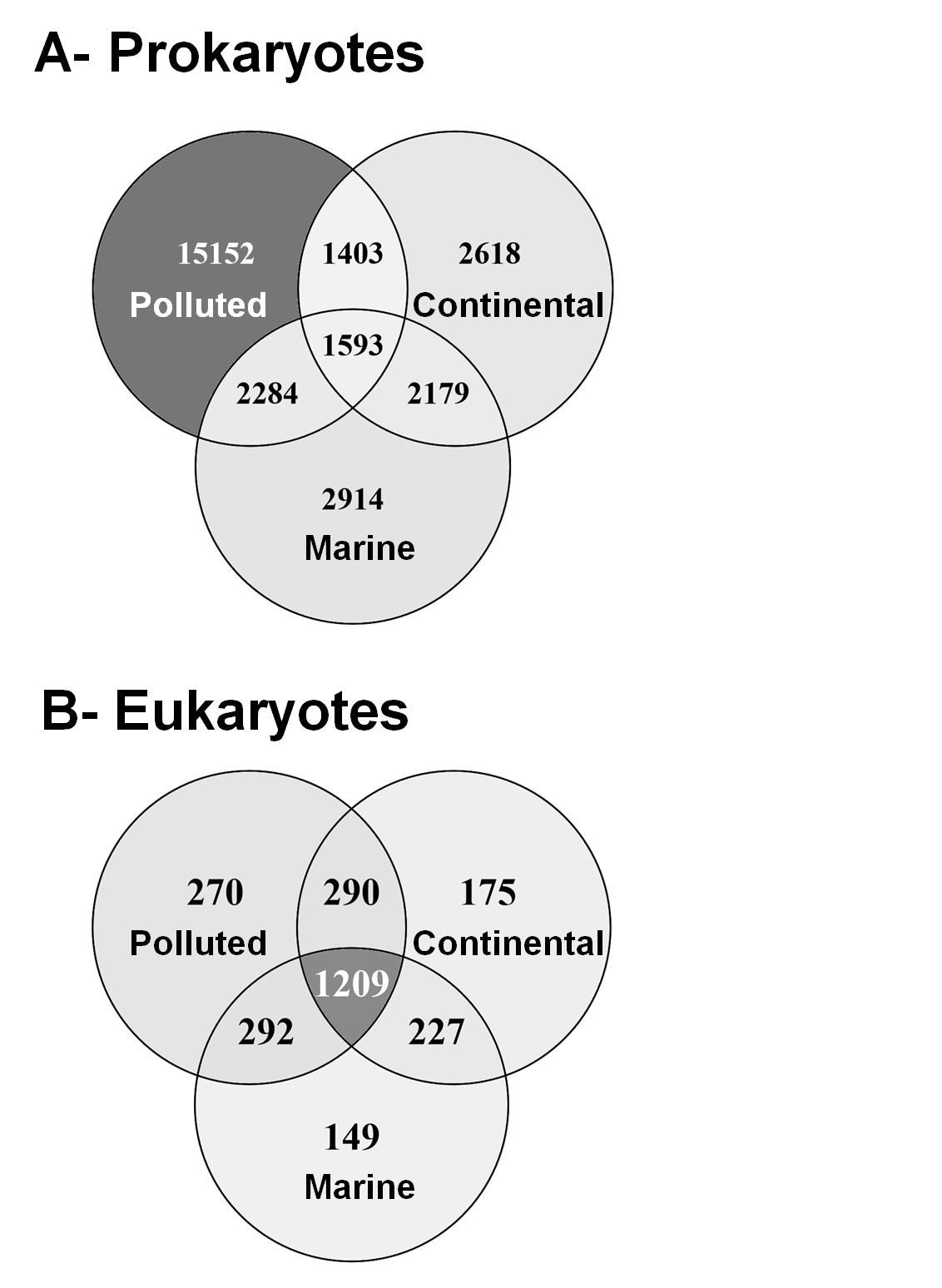

Supplement: S4 Fig — Venn diagrams depicting similarities and singularities of the 3 samples at the OTU0.03 level for prokaryotes (A) and OTU0.05 for eukaryotes (B). (TIF) [file pone.0182869.s007.tif]

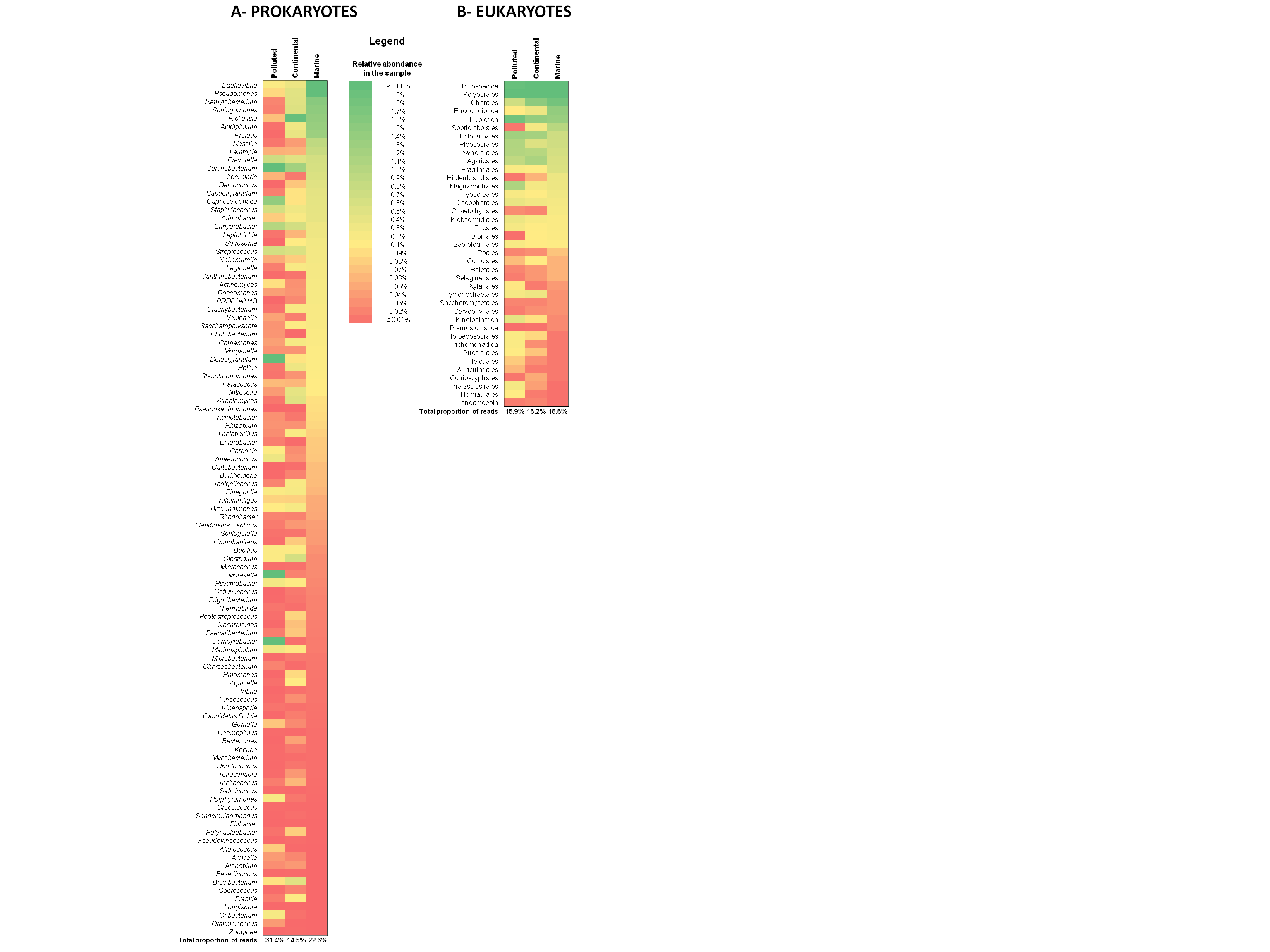

Supplement: S5 Fig — Relative abundance of shared prokaryotic genera (A) and eukaryotic orders (B). (TIF) [file pone.0182869.s008.tif]

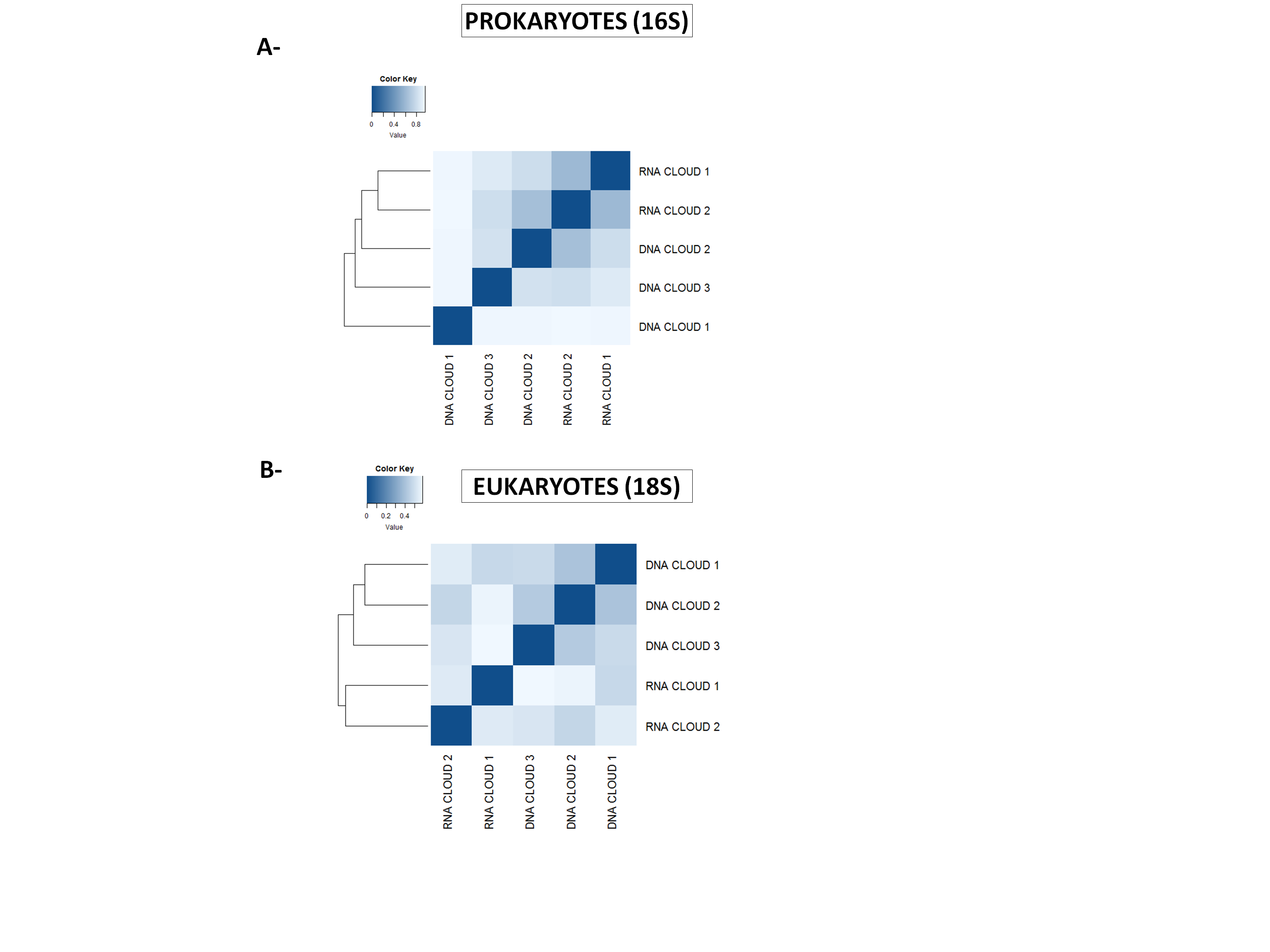

Supplement: S6 Fig — Bray-Curtis similarity matrices between the different sets of sequence of prokaryotes (A) and eukaryotes (B). (TIF) [file pone.0182869.s009.tif]

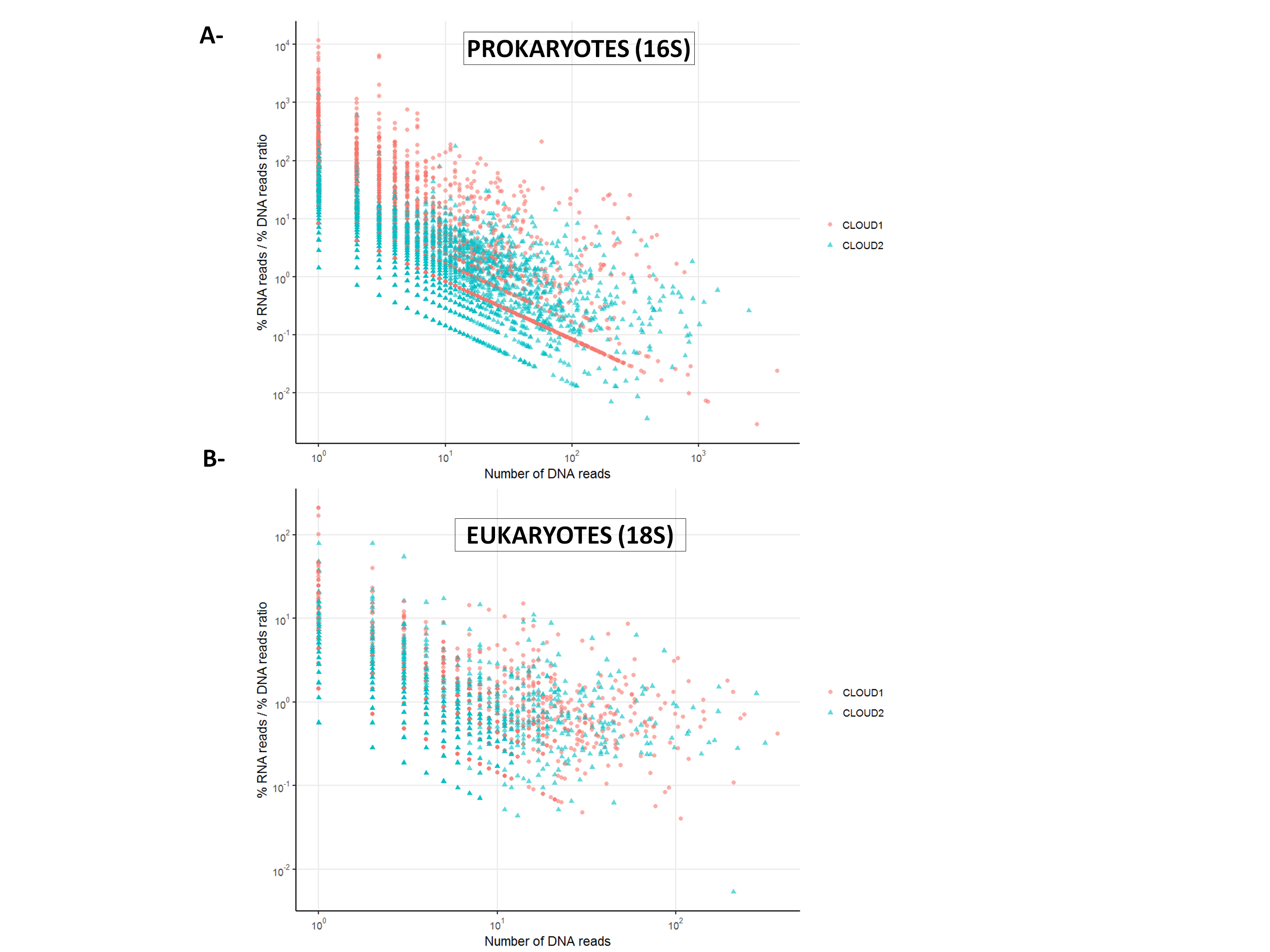

Supplement: S7 Fig — (TIF) [file pone.0182869.s010.tif]

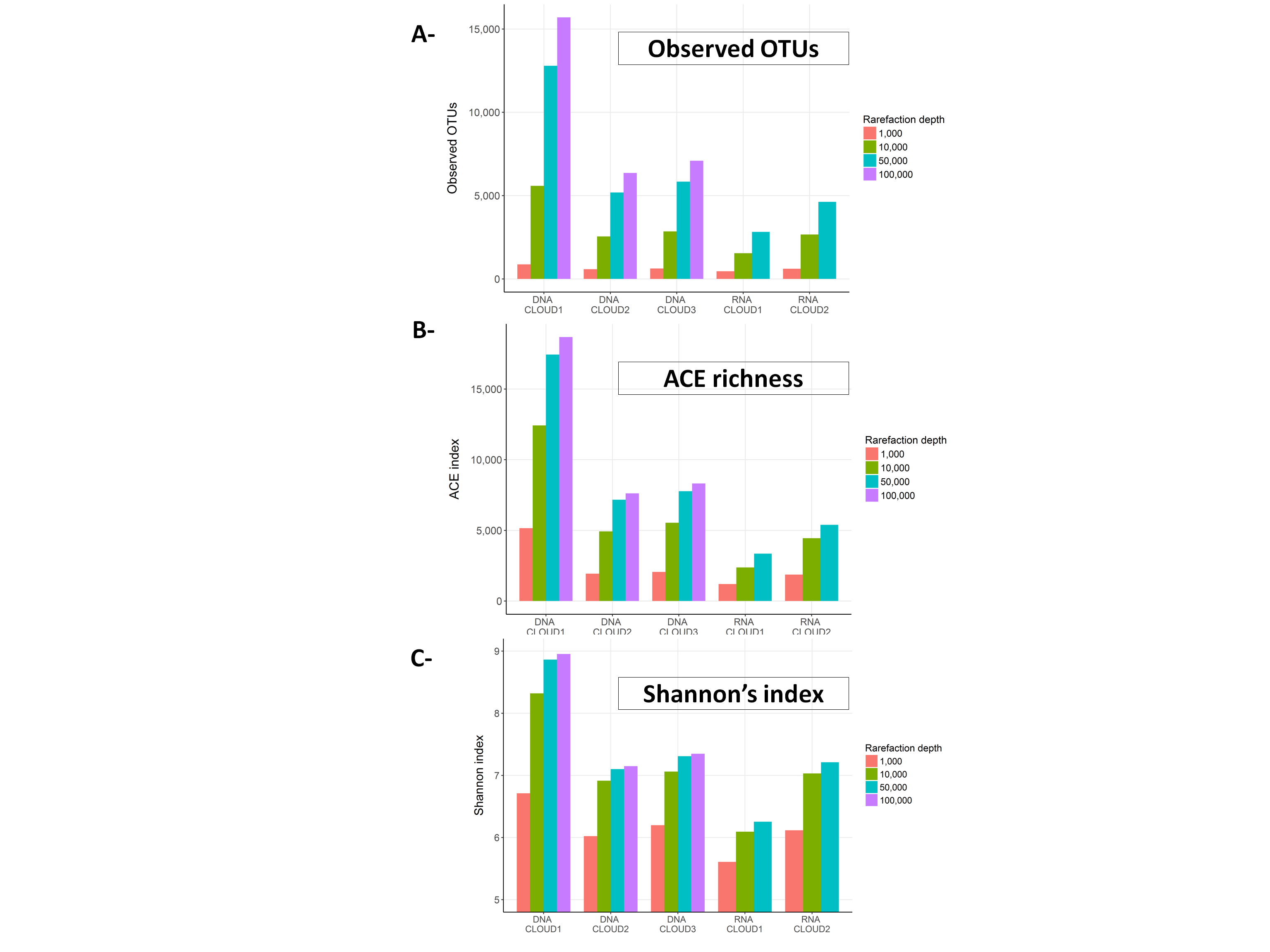

Supplement: S8 Fig — (TIF) [file pone.0182869.s011.tif]

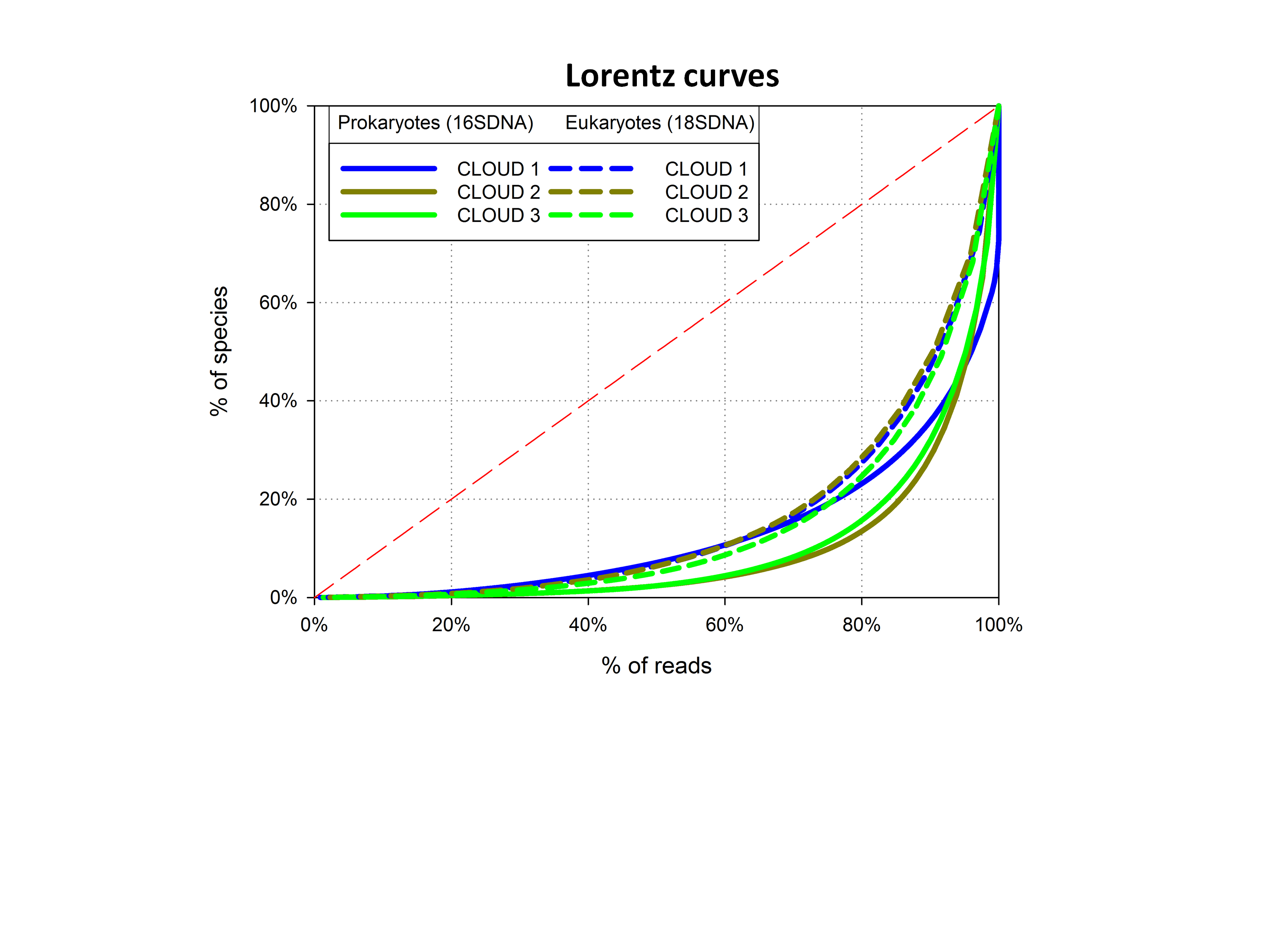

Supplement: S9 Fig — (TIF) [file pone.0182869.s012.tif]
